# Evaluation of Static and Dynamic Pupil and Light Sensitivity to a Single Drop of Various Concentrations of Low-Dose Atropine (0.01%, 0.025%, and 0.05%)

**DOI:** 10.3390/life15020278

**Published:** 2025-02-11

**Authors:** Muteb K. Alanazi, Abdulmalik Almansour, Sarah S. Almutairi, Ahmad Alharbi, Mohammed S. Alhazmi, Ali Almustanyir, Basal H. Altoaimi, Meznah S. Almutairi, Mona M. Alamri, Maria Liu

**Affiliations:** 1 Optometry Department, College of Applied Medical Sciences, King Saud University, Riyadh 11362, Saudi Arabia; 441100366@student.ksu.edu.sa (A.A.); 441200446@student.ksu.edu.sa (S.S.A.); ahmaalharbi@ksu.edu.sa (A.A.); malhazmyi@ksu.edu.sa (M.S.A.); aalmustanyir@ksu.edu.sa (A.A.); baltoaimi@ksu.edu.sa (B.H.A.); mzalmutairi@ksu.edu.sa (M.S.A.); malamrie@ksu.edu.sa (M.M.A.); 2 School of Optometry, University of California, Berkeley, CA 94720, USA; marialiu@berkeley.edu

**Keywords:** low-dose atropine, pupil, light sensitivity, dynamic pupil

## Abstract

This study aimed to evaluate the static and dynamic pupil changes, and light sensitivity following a single dose of low-dose atropine at concentrations of 0.01%, 0.025%, and 0.05% over a 24 h period. Healthy young adults (20–22 years; n = 25) participated in this randomized, double-blind study. Each participant received one of three atropine concentrations in a masked fashion. Baseline mesopic and dynamic pupil sizes were measured at various post-instillation intervals (5 min, 30 min, 1 h, 2 h, 4 h, and 24 h). A minimum 48 h washout period was observed between treatments. Subjective light sensitivity was assessed using the Visual Light Sensitivity Questionnaire (VLSQ-8) at 24 h. All atropine concentrations caused significant pupil dilation (*p* < 0.001), with the 0.05% concentration producing the greatest dilation (peak mesopic size: 7.4 mm, *p* < 0.001) and the slowest recovery at 24 h (6.4 mm, *p* < 0.001). The dynamic pupil constriction range was most restricted with 0.05% (1.7 mm, *p* < 0.05), compared to 0.025% (2.2 mm) and 0.01% (2.6 mm). Subjective symptoms, including light sensitivity and glare, followed a dose-dependent pattern (*p* < 0.05). In 60% participants, 0.05% caused the most symptoms, while in 70% participants, 0.01% caused the least. Despite significant pupil dilation, the pupil center coordinates did not shift significantly along the horizontal or vertical axes (*p* > 0.05). Low-dose atropine induced dose-dependent pupil dilation and light sensitivity; 0.05% atropine caused the most pronounced effects. These findings underscore the importance of tailoring the atropine dosage to balance its efficacy and tolerability. Further studies are needed to explore the long-term impact of repeated dosing on pupillary behavior and subjective symptoms.

## 1. Introduction

Currently, approximately one-third of the world’s population is myopic, a prevalence projected to reach 50% by 2050, affecting nearly five billion people worldwide [1]. This alarming rise in myopia prevalence represents more than a refractive inconvenience; it is a significant public health concern that could lead to substantial vision loss. The risks of vision-threatening conditions, including myopic maculopathy, cataracts, retinal detachment, and glaucoma [2] have been associated with myopia [3].

These complications are particularly pronounced in individuals with moderate (spherical equivalent ≤−3.0 to >−6.0 D) and high myopia (spherical equivalent ≤−6.0 D). The earlier the onset of myopia in childhood, the greater the likelihood of progression to severe forms, thereby increasing the lifetime risk of vision loss and economic burden [4,5]. Thus, early and effective management of myopia progression is crucial.

Several myopia control strategies have been investigated, demonstrating effectiveness rates ranging from 10 to 77%, depending on patient-specific factors, such as age, baseline refractive error, and treatment adherence. Myopia management approaches generally fall into two categories: optical treatments (e.g., overnight orthokeratology, peripheral defocus spectacle lenses, and soft multifocal contact lenses) and pharmaceutical interventions (e.g., atropine eye drops). Atropine eye drops are among the most widely studied and used options due to their effectiveness in slowing axial elongation and refractive error progression. However, higher concentrations of atropine (1% and 0.5%) are associated with significant side effects, including photophobia, near-vision blurring, and a higher risk of myopic rebound upon cessation. These disadvantages have shifted the focus toward low-dose atropine (≤0.05%), which still offers clinically meaningful efficacy with fewer side effects [6,7,8,9].

Low-dose atropine concentrations, particularly 0.01% and 0.025%, have gained widespread acceptance due to their ability to minimize pupil dilation and accommodation impairment compared to higher doses. Studies suggest that atropine concentrations of up to 0.02% cause negligible photophobia and blurring. Atropine at 0.01% remains the preferred starting concentration because of its excellent tolerability [10], though its myopia control efficacy is lower compared to 0.025% and 0.05% concentrations [11]. Despite these advantages, limited research has investigated the effects of low-dose atropine on static and dynamic pupillary behavior and light sensitivity across different concentrations. Understanding these effects is critical, as pupil size and responsiveness to light directly influence visual comfort, functional vision, and treatment adherence. Additionally, while higher concentrations of atropine are known to cause more pronounced physiological effects, there are limited data on pupil dynamics and subjective light sensitivity symptoms at low-dose concentrations of atropine. This information is essential for optimizing dosing strategies and balancing tolerability with clinical efficacy.

This study aimed to evaluate static and dynamic pupil changes and subjective light sensitivity over a 24 h period following a single application of low-dose atropine at concentrations of 0.01%, 0.025%, and 0.05% in healthy young adults. By investigating the physiological and subjective effects of these low-dose atropine concentrations, the findings contribute to tailoring atropine use to individual patient needs; this addresses tolerability concerns in myopia control and other clinical applications.

## 2. Materials and Methods

This randomized, blinded study enrolled 25 healthy young adults (15 males and 10 females) aged 20–22 years, all with no history of ocular or systemic diseases. Participants were screened for normal ocular health through comprehensive eye examinations, including best-corrected visual acuity (BCVA), slit-lamp biomicroscopy, and dilated fundus examination.

Participants were randomly assigned to receive a single drop of either 0.05%, 0.025%, or 0.01% atropine in both eyes. Both participants and examiners were masked to the assigned concentrations. Baseline static and dynamic pupil size measurements were taken before drop instillation. Mesopic and dynamic pupil responses were measured in an environment with an ambient light level of 20 lux using a MYAH (Topcon Healthcare Inc., Tokyo, Japan) at specific intervals: immediately after atropine instillation, and at 5 min, 30 min, 1 h, 2 h, 4 h, and 24 h. To control for diurnal variations, all baseline pupil measurements and atropine drop instillations for the three doses were conducted between 8:00 a.m. and 10:00 a.m.

Self-reported light sensitivity associated with pupil dilation was assessed at the 24 h time point for each concentration using the validated Visual Light Sensitivity Questionnaire (VLSQ-8) [12]. A 48 h washout period was implemented between doses to minimize carryover effects. This period starts 24 h after the drop instillation of the previous dose, ensuring a minimum of 36 h between atropine administrations. Previous studies on low-dose atropine, including 0.01%, have indicated that significant pupil changes and symptoms generally resolve within 18–24 h, supporting the adequacy of this interval for eliminating any residual effects [13].

This study was conducted in accordance with the guidelines of the Declaration of Helsinki and received approval from the Institutional Review Board of the College of Medicine, King Saud University (E-22-6783). Informed consent was obtained from all participants after the nature and purpose of the study were described.

Preservative-free atropine sulfate eye drops (0.01%, 0.025%, and 0.05%) were aseptically compounded by ImprimisRx (Irvine, CA, USA) and provided in single-dose vials. Each vial contained 5 mL of solution, which was stable for 6 months and stored at room temperature.

Statistical analysis included a summary of demographic and ocular characteristics, such as age, spherical equivalent (SE), axial length, best-corrected visual acuity, pupil size, and amplitude (Table 1). Continuous measurements were presented as mean ± standard deviation.

Repeated-measures analysis of variance (ANOVA) was employed to evaluate changes in mesopic and dynamic pupil sizes over the 24 h period. In this design, atropine concentrations (0.01%, 0.025%, and 0.05%) and eye (left or right) were considered within-subject factors, meaning that each participant was exposed to all three atropine concentrations, and pupil measurements were collected for both eyes of the same individual, allowing the direct comparison of effects across the different concentrations and between eyes within the same participant. Additionally, interactions between atropine concentration and eye were analyzed to determine whether the effects of atropine concentration differed significantly between the two eyes. Group comparisons were also performed using repeated-measures ANOVA, with post hoc multiple comparisons adjusted using the Bonferroni correction to control for Type I errors. Statistical significance level was set at 5% (*p* < 0.05). All statistical analyses were conducted using IBM SPSS Statistics version 29 (IBM Corp., Armonk, NY, USA).

## 3. Results

**Figure 1:**
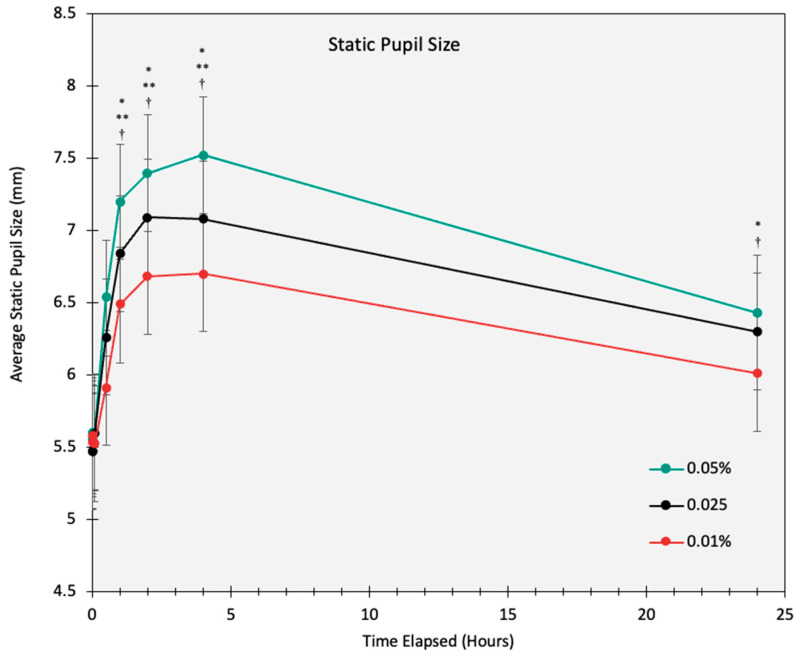
The static mesopic pupil size (mm) over a 24 h period for 0.01%, 0.025%, and 0.05% atropine concentrations. Error bars represent the standard error of the mean (SEM). An asterisk (*) indicates a significant difference in pupil size with 0.05% compared to 0.01% (*p* < 0.05) with adjustment for multiple comparisons (Bonferroni). A double asterisk (**) indicates a significant difference in pupil size with 0.05% compared to 0.025% (*p* < 0.05) with adjustment for multiple comparisons (Bonferroni). A dagger (†) indicates a significant difference in pupil size with 0.025% compared to 0.01% (*p* < 0.05) with adjustment for multiple comparisons (Bonferroni).

The pupil size data were collected from both eyes. No significant interocular differences were found between the two eyes (*p* > 0.05) for the main and interaction effects in the static and dynamic pupil size measurements. Thus, only data from the right eye were reported. The study demonstrated significant pupil dilation across all concentrations of low-dose atropine, with effects varying based on the dose. At baseline, the average mesopic pupil sizes were similar across concentrations, measuring 5.34 ± 0.8 mm, 5.35 ± 0.8 mm, and 5.43 ± 0.8 mm for 0.01%, 0.025%, and 0.05%, respectively. At 30 min post-instillation, mesopic pupil sizes increased to 6.2 mm, 6.4 mm, and 6.7 mm for 0.01%, 0.025%, and 0.05%, respectively. Peak dilation occurred at 4 h, with mean pupil sizes reaching 7.4 ± 0.6 mm, 7.1 ± 0.5 mm, and 6.7 ± 0.5 mm for 0.05%, 0.025%, and 0.01%, respectively. Partial recovery was observed by 24 h; however, mean pupil sizes remained elevated compared to baseline, measuring 6.4 mm, 6.3 mm, and 6.0 mm, respectively (Figure 1).

**Figure 2:**
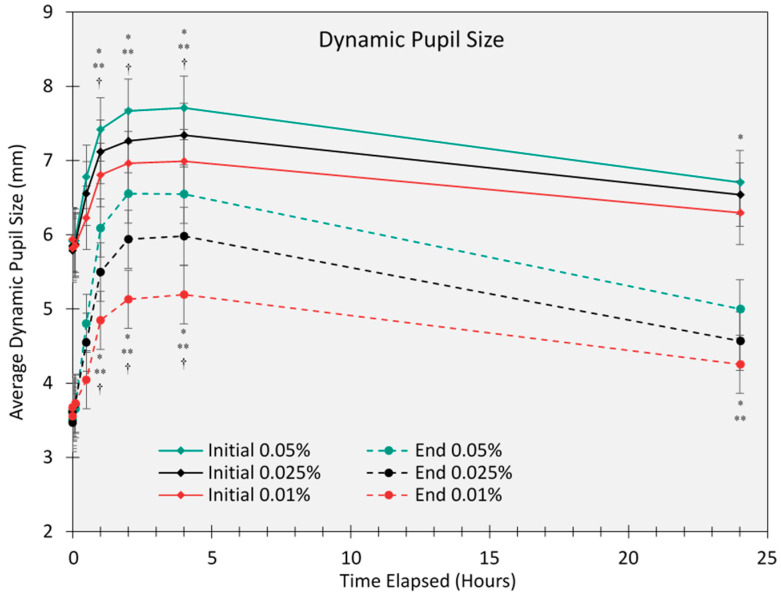
The initial (constriction to a light flash) and end (maximum re-dilation after a light flash) pupil size (mm) over a 24 h period for 0.01%, 0.025%, and 0.05% atropine concentrations. Error bars represent the standard error of the mean (SEM). An asterisk (*) indicates a significant difference in pupil size between 0.05% and 0.01% (*p* < 0.05), adjusted for multiple comparisons (Bonferroni). A double asterisk (**) indicates a significant difference in pupil size between 0.05% and 0.025% (*p* < 0.05), adjusted for multiple comparisons (Bonferroni). A dagger (†) indicates a significant difference in pupil size between 0.025% and 0.01% (*p* < 0.05), adjusted for multiple comparisons (Bonferroni).

Dynamic pupil responses, measured as the difference between maximum and minimum pupil sizes, revealed a dose-dependent reduction in the constriction range. The smallest dynamic pupil range was observed with 0.05% atropine (1.7 mm), compared to 2.2 mm for 0.025% and 2.6 mm for 0.01% (Figure 2). The analysis also revealed that there were no significant differences between male and female participants in static pupil size, minimum dynamic pupil size, or maximum dynamic pupil size, either as a main effect or in interaction with atropine concentration (all *p* > 0.05).

At baseline, the pupil center was displaced approximately 0.24 ± 0.014 mm nasally and 0.1 ± 0.09 mm inferiorly. Following the instillation of all three low-dose atropine concentrations, the pupil center remained consistently displaced nasally and inferiorly over the 24 h period. No significant shift in the pupil center was observed over time or when the three atropine concentrations were compared (*p* > 0.05).

Subjective light sensitivity scores collected using the Visual Light Sensitivity Questionnaire (VLSQ-8), demonstrated a clear dose-dependent trend. Participants reported significantly higher overall symptom scores for 0.05% atropine (20.7 ± 5.9) compared to 0.025% (16.8 ± 5.5) and 0.01% (13.2 ± 4.7). Specific symptoms, such as glare, blurred vision, and outdoor daylight sensitivity, were most pronounced with 0.05% atropine (Table 2). Participants were also asked to guess the concentration that caused the most and least symptoms after trying all three doses. Despite being blinded to the atropine concentration, 63% (15 participants) correctly identified 0.05% as causing the most symptoms, and 71% (17 participants) correctly identified 0.01% as causing the least symptoms, further supporting the subjective nature of dose-dependent symptom perception (Table 3).

A significant negative correlation was found between the overall symptom scores and the magnitude of change in dynamic pupil size at 1, 2, and 4 h post-instillation, with Pearson correlation coefficients of −0.298, −0.345, and −0.280, respectively (all *p* < 0.05). These associations indicate that larger dynamic pupil changes are associated with lower symptom scores, suggesting an inverse relationship between pupil adaptability and symptom severity.

## 4. Discussion

This study highlights the concentration-dependent effects of low-dose atropine on pupillary behavior, light sensitivity, and subjective visual symptoms. The significant pupil dilation observed at all concentrations aligns with atropine’s pharmacological inhibition of parasympathetic activity. Despite substantial changes in pupil size, the lack of significant shifts in pupil center coordinates along the horizontal (X) and vertical (Y) axes suggests that atropine-induced dilation does not alter the spatial alignment of the pupil. This finding is clinically relevant, as significant displacement of the pupil center could affect higher-order optical aberrations and visual function.

Dynamic pupil behavior demonstrated a dose-dependent reduction in the constriction range, with 0.05% atropine causing the most pronounced impairment. This suggests that higher concentrations not only prolong dilation, but also hinder the pupil’s ability to respond dynamically to changing light conditions. While this effect was less pronounced at lower concentrations, it underscores the need for caution when prescribing higher doses, particularly in patients requiring good photopic vision.

Subjective light sensitivity data revealed that higher atropine concentrations resulted in more pronounced visual symptoms, such as glare and discomfort. Interestingly, participants were able to accurately identify the concentration causing the most symptoms (0.05%) and the least symptoms (0.01%) in most cases despite being blinded to the concentrations. This finding reinforces the subjective impact of dose-dependent side effects and highlights the importance of patient education regarding potential symptoms during atropine therapy. While higher concentrations may offer greater efficacy for myopia control, the associated symptoms could reduce adherence, particularly in pediatric populations.

In addition to directly antagonizing muscarinic receptors, atropine’s effects on pupil dynamics and light sensitivity are affected by other pharmacological factors. The interaction of atropine with melanin in the iris can modulate its duration of action, as dark-brown irises with high melanin content bind more atropine molecules, potentially delaying its release into intraocular tissues. This can result in a slower onset but prolonged duration of action. Conversely, lighter-colored irises with lower melanin content may allow faster atropine penetration but shorter drug retention, resulting in a quicker yet shorter-lived effect. Furthermore, the transcorneal absorption and systemic clearance of atropine contribute to individual variability in its pharmacodynamic profile. These factors show the complexity of atropine’s effects and highlight the importance of considering both pharmacological mechanisms and individual characteristics when designing personalized atropine regimens [14,15].

This study’s findings align with those of Tran et al. (2024), who tested the daily administration of three low-dose atropine concentrations (0.01%, 0.02%, and 0.03%) in children for 2 weeks. Their results demonstrated a dose-dependent increase in pupil dilation with low-dose atropine, with higher concentrations inducing greater dilation, and more eyes experiencing pupil dilation exceeding 3 mm [16]. In contrast, the current study found that a single drop of low-dose atropine caused pupillary dilation lasting up to 24 h. Specifically, the proportion of eyes showing at least a 1 mm increase in mesopic pupil size 24 h after post-instillation was 16%, 68%, and 76% for 0.01%, 0.025%, and 0.05% concentrations, respectively. Notably, no eyes in this study exhibited pupil dilation exceeding 3 mm at the 24 h time point, suggesting that the greater pupil dilation observed in Tran et al.’s study is likely due to the cumulative effect associated with repeated daily doses over a 2-week period.

Kaymak et al. demonstrated significant pupil dilation under both photopic and mesopic conditions after a single drop of lower-concentration atropine (0.01%, 0.005%, and 0.001%). Similar to the current study, the effect of pupil dilation in Kaymak et al.’s study persisted for at least 24 h, with partial recovery observed during this period, even at much lower concentrations [17]. Additionally, Kaymak et al. reported increased variability in pupil size following a single drop, potentially influenced by iris pigmentation or atropine penetration variability. In contrast, the current study did not show any significantly increased variability, likely because all participants were Middle Eastern with dark brown irises. This homogenous population may have reduced variability and allowed for clearer correlations between pupil dynamics and subjective symptoms.

The significant correlation observed between the total symptom scores and the magnitude of dynamic pupil size changes at 1, 2, and 4 h post-instillation can be attributed to the critical role of dynamic pupil behavior in adapting to varying light conditions. Dynamic pupil size changes reflect the pupil’s ability to respond to light stimuli, regulating retinal light exposure, and consequently mitigating photosensitivity symptoms. This relationship highlights the functional importance of dynamic pupil adaptability in maintaining visual comfort, particularly in the context of atropine-induced dilation.

No significant correlation was observed at earlier time points (before 1 h), likely because the drops had not yet induced maximal dilation. Similarly, no significant correlation was observed at the 24 h mark, as the drops’ effects on pupil size had begun to wear off, reducing its impact on the photosensitivity symptom score. These findings align with the physiological timeline of atropine’s pharmacodynamics, where the most significant dilation typically begins approximately 1 h post-instillation and peaks at approximately 4 h under both mesopic and dynamic conditions.

The data further demonstrated that low-dose atropine affected both mesopic and dynamic pupil behavior, with the largest dilation occurring between 1 and 4 h post-administration. This highlights a critical period where patients are most likely to experience photosensitivity symptoms, correlating directly with the magnitude of dynamic pupillary changes. These results emphasize the need to address dynamic pupil effects when designing more tolerable atropine regimens, as reducing dynamic changes could help alleviate symptoms and improve adherence. These findings align with those of Cooper et al., who identified 0.02% atropine as the maximum concentration at which no significant symptoms were produced [10].

The myopia inhibition mechanisms of atropine remain unclear, and accommodation-related effects have been ruled out [18]. Atropine mimics light stimulation by enhancing nitric oxide and dopamine release and increasing contrast sensitivity at intermediate spatial frequencies [19,20,21,22]. Atropine likely does not act through muscarinic receptors, as the concentrations required for myopia suppression exceed those needed to saturate these receptors. Atropine also binds to alpha-adrenergic receptors, which may contribute to its effects. A 0.01% atropine drop is estimated to produce vitreal concentrations exceeding the M4 receptor half-saturation threshold, while lower doses (0.005% or 0.001%) approach this threshold. Although daily atropine use may lead to accumulation due to its prolonged effects on pupil size, this is less likely at lower doses. Further studies are needed to confirm whether accumulation occurs and its impact on pupillary behavior during prolonged application.

This study had some limitations. First, the sample size was small and limited to only young adults, which may limit the generalizability of the findings. While the randomized crossover design lessened variability by allowing each participant to serve as their own control, larger sample sizes are needed in future studies to validate these results and strengthen the conclusions drawn. Future studies should investigate pediatric populations with larger sample sizes. Second, all participants were Middle Eastern with dark-brown irises. Iris pigmentation may influence interocular absorption and the duration of action of low-dose atropine. Dark-colored irises with high melanin content bind more atropine molecules than light-colored irises, potentially delaying drug release into intraocular tissues [23,24]. This may be associated with a more gradual onset of action, but may have an advantage in prolonging the effect of the drug because atropine is gradually released from melanin stores. In contrast, lighter-colored irises containing less melanin may allow quicker atropine penetration and shorter drug retention, thereby producing a quicker but probably shorter duration of action. This variability could explain individual differences in pupil size changes and symptom duration following low-dose atropine, depending on iris pigmentation. These findings could have important implications for optimizing atropine regimens, especially among different populations. Expanding the study to include participants with diverse iris colors, particularly lighter-colored irises, would provide a broader understanding of how pigmentation influences atropine’s effects, including pupil dynamics and symptom variability. Third, the accommodative response following a single low dose of atropine was not measured in this study, which limits our ability to fully understand the functional impact of atropine on near vision and how it may contribute to subjective symptoms like blurred vision. Lastly, a formal statistical power calculation was not performed before the study, possibly affecting the generalizability of the findings. While significant results were found, future studies with larger sample sizes and pre-calculated power analyses are needed to validate and build upon these findings.

These results also emphasize the need for individualized treatment strategies. Lower concentrations, such as 0.01%, may provide a more tolerable option for patients prioritizing visual comfort, whereas higher doses may be reserved for those requiring maximal myopia control efficacy. Clinicians should also consider alternative strategies, such as alternate-day dosing, to minimize side effects. Future research should investigate the long-term effects of daily low-dose atropine on pupil dynamics and subjective symptoms. Additionally, studies assessing the impact of repeated dosing on static and dynamic pupil changes would provide further insights into atropine-induced dilation and the associated light sensitivity symptoms.

## 5. Conclusions

This study demonstrated the dose-dependent effects of low-dose atropine (0.01%, 0.025%, and 0.05%) on static and dynamic pupil behavior, as well as subjective light sensitivity, in a controlled cohort of young adults. Significant pupil dilation was observed across all concentrations, with the 0.05% dose producing the most pronounced and sustained effects. Dynamic pupil responses revealed a dose-dependent reduction in constriction range, suggesting that higher atropine concentrations impair the pupil’s ability to adapt to changing light conditions. Subjective symptoms, including glare and light sensitivity, followed a similar dose-dependent pattern, underscoring the trade-off between therapeutic efficacy and tolerability.

These findings highlight the need for individualized atropine dosing strategies that balance efficacy with minimizing adverse effects, particularly for long-term use in myopia control. While higher concentrations demonstrate greater physiological effects, they are associated with increased visual symptoms, which may impact treatment adherence, especially in pediatric populations. The study establishes foundational data on short-term of a single drop of low dose atropine concentrations, providing a foundation for future research exploring its use in pediatric populations and long-term treatment to improve both clinical outcomes and patient comfort.

## Figures and Tables

**Table 1:** Demographic and ocular characteristics of the study participants (N = 25).

	Mean ± SD	Range
Age (years)	21.1 ± 0.7	20–22
Best-corrected visual acuity (LogMAR)	0.013 ± 0.05	−0.1–+0.1
Spherical equivalent (D)	−1.3 ± 2.2	−6.7–+2.1
Axial length (mm)	24.2 ± 1.2	22.05–26.9

**Table 2:** Visual Light Sensitivity Questionnaire (VLSQ-8) scores for individual items across atropine concentrations (0.01%, 0.025%, and 0.05%). Data are presented as mean ± standard deviation (SD).

Questionnaire Item	0.01%	0.025%	0.05%
	Mean ± SD	Mean ± SD	Mean ± SD
Outdoor Daylight	1.9 ± 1	2.7 ± 1.5 *	3.2 ± 1.1 *
Glare	1.5 ± 0.8	2 ± 1.1	2.6 ± 1.4 *
Flickering lights	1.6 ± 0.8	2.2 ± 1.2	2.6 ± 1.3 *
Severity	1.7 ± 0.8	2.2 ± 1	2.9 ± 1 *†
Headache	1.5 ± 0.9	1.6 ± 0.8	2 ± 0.8
Blur vision	1.7 ± 0.9	2.5 ± 1.1 *	2.9 ± 1.4 *
limitations	1.7 ± 0.9	2.1 ± 1.2	2.2 ± 1.2
Dark glasses	1.8 ± 1.4	1.7 ± 1.2	2.4 ± 1.5
Overall Score	13.2 ± 4.7	16.8 ± 5.5 *	20.7 ± 5.9 *†

An asterisk (*) indicates a significant difference compared to 0.01% (*p* < 0.05), and a dagger (†) indicates a significant difference compared to 0.025% (*p* < 0.05), both adjusted for multiple comparisons using the Bonferroni method.

**Table 3:** The number of participants who identified the atropine concentration based on experienced symptoms.

	Guessed Atropine Concentration Correctly	Guessed Atropine Concentration Incorrectly	Unable to Differentiate
Least symptoms (0.01% Atropine)	17	5	3
Most symptoms (0.05% Atropine)	15	8	2

## Data Availability

Data were collected and analyzed as part of this study to assess the effects of low-dose atropine on static and dynamic pupil behavior and light sensitivity. The data are available upon reasonable request.

## Abbreviations

The following abbreviations are used in this manuscript:

BCVA	best-corrected visual acuity
VLSQ-8	Visual Light Sensitivity Questionnaire
ANOVA	analysis of variance
SEM	standard error of the mean
